# Effective Outcome of HBOT as an Adjuvant Therapy in Patients Diagnosed with COVID-19 in a Tertiary Care Hospital – A Preliminary Study

**DOI:** 10.2478/jccm-2022-0008

**Published:** 2022-08-12

**Authors:** Thiruppathy Palaniappan, Asif Shaikh, Navamani Kirthika

**Affiliations:** 1 Medway Hospital, Chennai, India

**Keywords:** COVID-19, hyperbaric oxygen therapy, infectious diseases, partial pressure, oxygen

## Abstract

**Introduction:**

Hyperbaricoxygen therapy (HBOT) is breathing100% oxygen in pressurised chamber. This therapy ensures quick oxygen delivery to the bloodstream. In patients with severe COVID-19 pneumonia, progressive hypoxemia occurs. Oxygen therapy hasa significant role in its management.

**Aim of the study:**

The objective was to study the efficacy of hyperbaric oxygen therapy (HBOT) as adjuvant therapy for reducing the requirement of additional oxygen supplementationin patients with moderate to severe ARDS diagnosed with COVID-19.

**Methods:**

A single-centre prospective pilot cohort study was conducted ata tertiary care hospital from December 2020 to February 2021 over two months. Fifty patients with COVID-19 needingoxygen, satisfying the selection criteria, were included. Hyperbaricoxygen therapy wasgiven to all patients. The patient received30-45 minutes of hyperbaric oxygen with 15 minutes of pressurizing and depressurizing at 2.0 atmosphere absolute (ATA) with or without airbrakesas per the critical care team. Oxygen requirement, PaO2, andcondition at discharge were considered as primary outcome variables.

**Results:**

Among the 50 participants studied, the mean age was 53.64±13.26 years. Out of 50 participants, 49(98.00%) had PaO2≤80 mmHg, and one (2.00%) had >80 PaO2. All the participants 50(100%) had PaO2 as 90 mmHg after three sittings.

**Conclusion:**

This studyshows promising results in using HBOT to overcome respiratory failure in COVID-19. HBOT reduced the need for oxygen by improving the oxygen saturation levels.

## Introduction

COVID-19 is the newest member of the betacoronavirus family, first identified inWuhan province, China, in2019. Severe acute respiratory syndrome coronavirus two (SARS CoV 2) was named severe acute respiratory syndrome two because of its similarities with the previous viruses [1].

COVID-19 is the second form of SARS, which previously causeda global pandemic to spread almost to 29 countries in 2002. The severity spectrum wasclassified into mild (81%), severe (14%), orcritical (5%) [2], where respiratory failure is the primary cause of death due to SARS virus [3]. Similarly, COVID-19 also affects the respiratory system and causes oxygen deprivation. The abnormal response of host’s immune system leads to a prolonged inflammatory response whichcausescytokine storm. Inrecent times, few cases had no symptoms of pneumonia, but the patient rapidly progressed and fell critically ill with respiratory failure. This can be caused due to alveolocapillary membrane disorder leading to oxygen delivery impairment causing hypoxia. This has been termed as silent or happy hypoxia [4].

Attention should be given to this condition because this leads to oxygen deprivation and leading to drop in partial pressure of oxygen. These patients need immediate oxygen therapy. Standard therapy of oxygen delivery for COVID-19 patients is complex due to the inflammation in the lungs caused by the virus, thereby limiting the oxygen uptake by the red blood cells. Due to this difficulty, hyperbaric oxygen therapy (HBOT) plays anadjuvant role in patients with silent hypoxia by its ability to rapidly increase dissolved oxygen levels in blood andpaythe oxygen debt. Within the chamber, the high-pressure oxygen is delivered through face masks and hoods. The pressure inside the chamber is usually about 250-280 kPa. The HBOT sessions need can be up to five and the duration varies from 45 to 300 min. Vigilant monitoring is essential during HBOT, and amenities for resuscitation, mechanical ventilation must be kept ready [5]. The potential risks and riskbenefit ratio of hyperbaric oxygen have often been underemphasized in therapeutic trials.In routine practise, HBOT is considered safe when the pressure if less than 300 kPa and duration is less than 120 minutes.

This is the first of its kind study done on the role of HBOT in COVID-19 patients. This paper focuses on the effect of HBOT in reducing the requirement of additional oxygen supplementationin moderate and severe COVID-19 patients admitted at a tertiary care hospital.

## Methods

A prospective pilot cohort study has conducted ata tertiary care hospital, from December 2020 to February 2021 over two months. The patients signed informed written consents. The critical care team selected fifty COVID-19 patients based on the selection criteria by convenient sampling technique. Patients included in the current study were those patients above 18 years of age and less than 85 years,who were within seven days of developing oxygen demand, positive SARS-CoV-2 RT-PCR, rapid antigen positive with HRCT score more than 20/40 CORADS, those with respiratory insufficiency inroom air i.e., SpO2 <90% or PaO2/ FiO2<300mmHg in room air with PaO2 based on arterial blood gas analysis of less than 80, patientsrequiring oxygen more than six liters/min, patients with one or more comorbidities like uncontrolled diabetes, hypertension, Coronary artery disease (CAD), obesity, hypothyroidism, Chronic kidney disease (CKD), chronic liver disease and patients with moderate or severe covid pneumonia. Patients with mild pneumonia and patients with HBOT contraindications such as claustrophobia, pneumothorax, pneumo-mediastinum, andear/sinus disease were not allowed in HBOT, known cases ofthe chronic pulmonary disease: severe emphysema or known case of pulmonary bullae, pregnancy, age above 85 years and patients on mechanical ventilation were excluded from the study.

Patients were screened for COVID-19 with help of rapid antigen test, RT-PCR and HRCT thorax. Were admitted in COVID-19specialty unit. All patients were treated according to their oxygen requirement and the lung involvement in HRCT thorax.

### Clinical criteria for giving oxygen therapy

1. Patient’s SPO2 less than 90% in room air
2. Breathlessness of class 3 as per NYHA.

### Clinical Criteria for starting antiviral therapy and steroid therapy

**Anti-viraltherapy**: InjRemedesvir (Cipla, India) 100mg* 2 vials as initial dose considering the patients renal profile (serum creatinine level), followed by 100mg once a day for 4 days and renal profile monitored every alternate day)

1. Patients aged more than 60yrs
2. Patients with comorbidities mentioned in inclusion criteria
3. Patients with CORADS score of more than 20 as per radiological evidence

### Criteria for steroid therapy

1. Patients with NYHA class 3 breathlessness
2. Oxygen requirement more than 2liters/min
3. Patients with comorbidities
4. Patients with high blood sugar levels were monitored while giving steroid therapy and treated accordingly with OHA or insulin.

Inflammatory markers checked for the patients wereinterleukin-6 (IL6), Lactate dehydrogenase (LDH), D dimer, C reactive protein (CRP), Serum ferritin and other markers such as tumor necrosis factor (TNF -alfa), interleukin (IL-8, IL-9). These markers were checked at regular intervals and patients with increased inflammatory markers were managed as per the protocol.

Cytokine storm was considered whenpatients had symptoms worsening rapidly even after treatment where inflammatory marker were severely deranged specifically CRP markedly elevated. It suggests, patient is progressing to sever form of illness which will involve multi organ damage.

### Criteria for HBOT

Patients with CORADS more than 20 requiring oxygenation showing progressive illness despite of conventional therapy which is antiviral and steroid therapy. And showing signs of increased inflammatory markers.Patients with comorbidities mentioned above and showing increasing inflammatory markers and rapidly progressing towards multiorgan dysfunction were selected for HBOT.

The study-selected participants were shifted to the COVID-19 unit and were assessed by the critical care team. The patients were taken to the hyperbaric unit by maintaining airborne precautions based on hospital protocol. All team members handling the patients wore complete personal protective equipment. Once the patient was placed inside the monoplane chamber, and the doors were locked, the patient was instructed to remove the PPE and masks.

The patient received 30 to 45 minutes of hyperbaric oxygen with 15 minutes of pressuring and depressurizing at 2.0 ATA with or without airbrakes per the hyperbaric physician.Electrocardiogram (ECG), pulseoximetry, and temperature monitoring were done at regular intervals inside the hyperbaric chamber. Noninvasive blood pressure monitoring (NIBP) was used safely inside the chambers.

HBOT was given in three sittings on alternate days. The need for oxygen was assessed at the baseline and compared with the need after three sittings of HBOT. PaO2 monitoring was done by ABG (arterial blood gas) monitoring 12^th^ hourly to monitor the further requirement of HBOT, and to note the significance of the treatment by observing the improvement in PaO2 levels; inflammatory markers were checked regularly to observe changes and response to treatment, daily chest x-rays were taken, monitoring of parameters like oxygen requirement, vitals monitoring.

### Statistical analysis

Oxygen requirement, PaO2, and condition at discharge were considered as the primary outcome variable. Descriptive statisticswas done as per the study objectives. Mean, 95% confidence interval (CI, lower and upper bounds), median, minimum and maximum, and percentage, were used to express the data. Categorical outcomes were compared between study groups using the McNemar test. Data was analyzed by using the coGuide software, V.1.03[6].

## Results

A total of 50 subjects were included in the final analysis.

The mean age was 53.64±13.26 years in the study population(ranged 29 to 82). Among the study population, 30(60%) participants were male, and the remaining 20(40%) were female. The mean c-reactive protein levels were 62.98 ± 23.12 mg/l, ranged from 56.41 to 69.55, the mean D dimer was 0.53 ± 0.1ng/ml, ranged from 0.50 to 0.56, the mean IL6 was 13.06 ± 9.07 pg/ ml, ranged from 10.48 to 15.64, Ferritin was normal for 48(96%) participants and all of them had normal lactate dehydrogenase and the mean CORAD score was 5.18 ± 0.56, ranged from 5.02 to 5.34. There were 4(8%) only with CORAD score 4 and CORAD score 5 and 6 reported in 66%, 26% respectively. (Table 1).Clinical presentation of the patients were fever, cough, which developed over period of 3 days and few had myalgia, vomiting and diarrhea.

**Table 1:** Summary of baseline parameter (N=50)

Parameter	Summary
Age (in years)	53.64 ± 13.26 (ranged 29 to 82)

Gender	
Male	30 (60.00%)
Female	20 (40.00%)

Lab parameter	
C-reactive protein levels (in mg/L)	62.98 ± 23.12 (ranged 56.41 to 69.55)
D Dimer (in ng/mL)	0.53 ± 0.1 (ranged 0.50 to 0.56)
IL-6 (in pg/mL)	13.06 ± 9.07 (ranged 10.48 to 15.64)
Ferritin (normal) (in ng/mL)	48 (96%)
Lactate dehydrogenase (normal) (in U/L)	50 (100%)
CORAD Score	5.18 ± 0.56 (ranged 5.02 to 5.34)

CORAD Score	
4 (High- abnormalities suspicious of COVID)	4 (8%)
5 (very high- Typical COVID)	33 (66%)

The majority of 70% had diabetic mellitus, followed by 60% had hypertension, 88% had moderate severity. The mean ATA depth was 1.7±0.11 in the study population(ranged 1.50 to 1.80). Among Tx time, 29(58.00%) were 45 min. All of them, 50(100%), were in room air condition at discharge. All of them 100% were reported pressure time and de-pressureas 15 min each respectively. Three sittings were considered for all the study participants. (Table 2)

**Table 2:** Summary of clinical and outcome parameter (N=50)

Parameter	Summary
Comorbidities	
Diabetic mellitus	35 (70%)
Hypertension	30 (60%)
Obesity	2 (4%)
Asthma	1 (2%)
Dyslipidaemia	1 (2%)
Hypothyroidism	1 (2%)

Severity of pneumonia	
Moderate	44 (88.00%)
Severe	6(12.00%)

ATA Depth	1.7 ± 0.11(ranged 1.50 to 1.80)

Tx Time (Minutes)	
45	29 (58.00%)
60	21 (42.00%)

Condition at discharge	
Breathing room air	50 (100%)

On day 1, for the majority of 36% of participants, we gave 15 liters of oxygen, followed by 26% participants with 13 liters of oxygen and 16% participants with 10 liters of oxygen. The majority of 90% participants showed improvement to room air condition after three sittings of HBOT. Out of 50 participants at admission,17(34%) hadPaO2 ≤60 mmHg,29(58%) had 61-70 mmHg, 3(6%) had 71-80 mmHg PaO2 and one (2.00%) had >80 mmHg PaO2. All the participants 50(100%) had PaO2 as 90 mmHg after 3 sittings of HBOT. (Table 3)

**Table 3:** Summary of oxygen requirement and PaO_2_ at day one and after three sittings of HBOT (N=50)

Parameter	Summary
Oxygen Requirement (per minute) Day1	
4 liters	2(4.00%)
6 liters	13(26.00%)
8 liters	6(12.00%)
10 liters	8(16.00%)
15 liters	18(36.00%)
15 liters to HFNC	3(6.00%)

Oxygen Requirement (per minute) After 3 Sittings of HBOT	
Room air	45(90.00%)
2 liters	2(4.00%)
4 liters	2(4.00%)
6 liters	1(2.00%)

PaO_2_ (mm Hg) (At admission)	
≤60	17(54.00%)
61-70	29 (58%)
71-80	3 (6%)
>80	1(2.00%)

PaO_2_ after three sittings (mm Hg)	
≤60	0 (0%)
61-70	0 (0%)
71-80	0 (0%)
≥90	50(100%)

Those study participants with oxygen requirement of 4 to 10 liters per minuteon day one, all 100% reported room air condition after three sittings. Out of 18participants with 15 litersof oxygen requirement at day one, two (11.11%) reported 4 liters, and one (5.56%) reported 2 liters of oxygen after three sittings. Among three participants at day one with HFNC,one (33.33%) reported 2 liters, 6 liters, and room air condition after three sittings. (Table 4)

**Table 4:** Comparison of oxygen requirement at day one and after three sittings (N=50)

Oxygen Requirement (per minute)	Oxygen Requirement (per minute) after 3 Sittings			
Day one	2 liters	4 liters	6 liters	Room air condition
4 liters (N=2)	0 (0%)	0 (0%)	0 (0%)	2 (100%)
6 liters (N=13)	0 (0%)	0 (0%)	0 (0%)	13 (100%)
8 liters (N=6)	0 (0%)	0 (0%)	0 (0%)	6 (100%)
10 liters (N=8)	0 (0%)	0 (0%)	0 (0%)	8 (100%)
15 liters (N=18)	1 (5.56%)	2 (11.11%)	0 (0%)	15 (83.33%)
15 liters to HFNC (N=3)	1 (33.33%)	0 (0%)	1 (33.33%)	1 (33.33%)

*No statistical test was applied- due to 0 subjects in the cells

## Discussion

This study’s significant findings showed improvement inthe partial pressure of oxygen after HBOT (PaO2 was reported as 90 mmHg after three sittings irrespective of the PaO2 at day one). The need for oxygen also gradually reduced after the HBOT sittings.

The prominent feature from COVID-19 patientsis hypoxia due to respiratory failure. However, recently, some report showed that COVID-19 patients were admitted to the hospital with lowoxygen saturation but without initial symptoms of dyspnoea. Guan reported that dyspnoea was only found in 18,7% of 1099 patients COVID-19 who were hospitalized, despite ratio PaO2/ FiO2, abnormal CT scans (86%), and use of supplemental oxygen (41%) [7]. Dr. Richard Levitan found the same result from Bellevue Hospital New York, who observed the blood oxygen levels of COVID-19 patients where they experienced arterial hypoxemia but did not show equivalent respiratory distress and did not even complain of dyspnea [8,9].

The current study findings show that the oxygen requirement of the patients was reduced once they were put on HBOT. Similar results have been reported worldwide. Two articles that have been published in China show that HBOT has the potential to be used in COVID–19 patients with pneumonia. The first publication was a case report of a patient with very severe symptoms who experienced respiratory failure (not intubated), and the symptoms improved after eightsittings of HBOT at 200 kPa for 95 minutes [10]. Second publicationwas a report of a patient on the ventilator with more severe disease symptoms with ARDS, who-was saved by using HBOT. Four other COVID-19 patients hadsevere symptoms with ground-glass opacities who could no longer be treated using standard oxygen masks, were treated with 3-5 sittings of HBOT and returned home [11] agent used for sedation, presence of a chest tube, need for vasopressor agents and tolerance and appearance of side effects. Finally, we compared the outcomes of patients according to the presence or absence of acute respiratory distress syndrome (ARDS). The same researchers also performed HBOT therapy on 20 other patients with milder symptoms and had the same successful outcome. Five patients who were not intubated had used oxygen for several days or even weeks with their pre-HBOT oxygen saturation of 70% (using oxygen mask). With daily HBOT administration, the patient experienced a persistent increase in oxygen saturation and improved symptoms the day after. With only 3-8 times HBOT, the patient can go through the hypoxic crisis phase of the disease and be discharged from the hospital. Researchers suggest that giving HBOT at the initial process of the disease can prevent worsening conditions, leading to significant morbidity and mortality of COVID-19 infection [10].

All airborne precautions control was followed in the current study, and all personnel used PPE to reduce cross-infection. Similarly, decreasing rates of cross-sections were observed in the study by Harch P et al. [10]. This makes HBOT an appropriate management modality, reducing the risk of transmission to health-care workers in the hospital setting.

Thibodeaux et al. [12] reported giving HBOT in 5 patients from April 13th to 20th with symptoms of tachypnoea and low oxygen saturation. The patient was given a high FiO, and the administration of HBOT is to avoid the use of mechanical ventilation. The therapy given was 2 ATA for 90 minutes nonstop in a monoplace chamber. Treatment was given 1-6 times. The result obtained was all patients were not on a ventilator, and there was a rapid improvement in tachypnoea and an increase in oxygen saturation. Within less than one week from the last therapy, three patients were discharged, and two were still being treated in a stable condition.

Similar to these reports, the current study showed that three sittings of HBOT for 30-45 minutes improved the patient’s oxygen levels (PaO2 was 90 mmHg after three sittings irrespective of the PaO2 at day one) and reduced the need for oxygen.

The limitation of the current study is its small sample size, and long-term follow-ups were not done. Studies involving large sample with the different patient profile is recommended in future.

Until now, COVID-19 has not been included in the list of indications for the use of HBOT. This is the right time to consider including COVID-19 as an indication for HBOT.

## Conclusion

Anti-inflammatory properties, high-pressure ambient and the use of 100% oxygen guarantee through HBOT, increased oxygen partial pressure in circulation and tissue despite decreased aerated pulmonary in COVID-19 patient with ARDS. With oxygen debt being ‘payed’ by HBOT, it will reverse tissue hypoxia. Hence, in clinical practice, HBOT can be considered in moderate and severe COVID-19 patients within seven days of developing oxygen demand.

## List of abbreviations

(HBOT): Hyperbaric Oxygen Therapy
(ATA): Atmosphere Absolute
(HRCT): High Resolution Computed Tomography
(CAD): Coronary Artery Disease
(CKD): Chronic Kidney Disease
(RTPCR): Reverse Transcriptase – Polymerase Chain Reaction
(NIBP): Non-Invasive Blood Pressure Monitoring
(CI): Confidence Interval
(IL-6): Interleukin-6
(LDH): Lactate Dehydrogenase
(CRP): C Reactive Protein
(TNF) Alpha: Tumour Necrosis Factor
